# The impact of the COVID-19 pandemic in the clinical assistance to breast cancer patients

**DOI:** 10.1007/s10552-023-01762-3

**Published:** 2023-08-05

**Authors:** Inês Ribeiro, Bárbara Peleteiro, José Luís Fougo

**Affiliations:** 1 Faculty of Medicine, University of Porto, Porto, Portugal; 2 Breast Center, Centro Hospitalar Universitário São João, Porto, Portugal; 3 EPI Unit, Institute of Public Health, University of Porto, Porto, Portugal; 4 Department of Surgery and Physiology, Faculty of Medicine, University of Porto, Porto, Portugal; 5 Laboratory for Integrative and Translation Research in Population Health, University of Porto, Porto, Portugal

**Keywords:** Breast cancer, COVID-19, Waiting time, Lymph node metastasis, Mammography, Early detection

## Abstract

**Purpose:**

We aimed to disclose the impact of the pandemic on breast cancer patients in a specialized breast cancer center (BCC).

**Methods:**

A total of 501 breast cancer patients with a first appointment in the BCC from April 1st, 2019 to March 31st, 2021 were divided into four consecutive periods of 6 months. Data from the homologous semesters was compared. Patients with an appointment in the BCC during the study period were eligible for the secondary aim of our study (BCC workload).

**Results:**

After the pandemic declaration (period 3), we found a decrease in the referral by screening programs (*p* = 0.002) and a reduction in the waiting time between the primary care referral and the first BCC appointment (*p* < 0.001). There were higher rates of palpable axillary nodes (*p* = 0.001), an increase in N stage 2 and 3 (*p* = 0.050), and a trend for primary endocrine therapy as the first treatment (*p* = 0.021) associated with higher rates of complete axillary node dissection (*p* = 0.030). In period 4, there were more outward diagnoses (*p* = 0.003) and a higher rate of surgery as the first treatment (*p* = 0.013).

**Conclusion:**

COVID-19 pandemic implied a more advanced nodal stage, which may be related to the delay in breast cancer screening.

**Graphical abstract:**

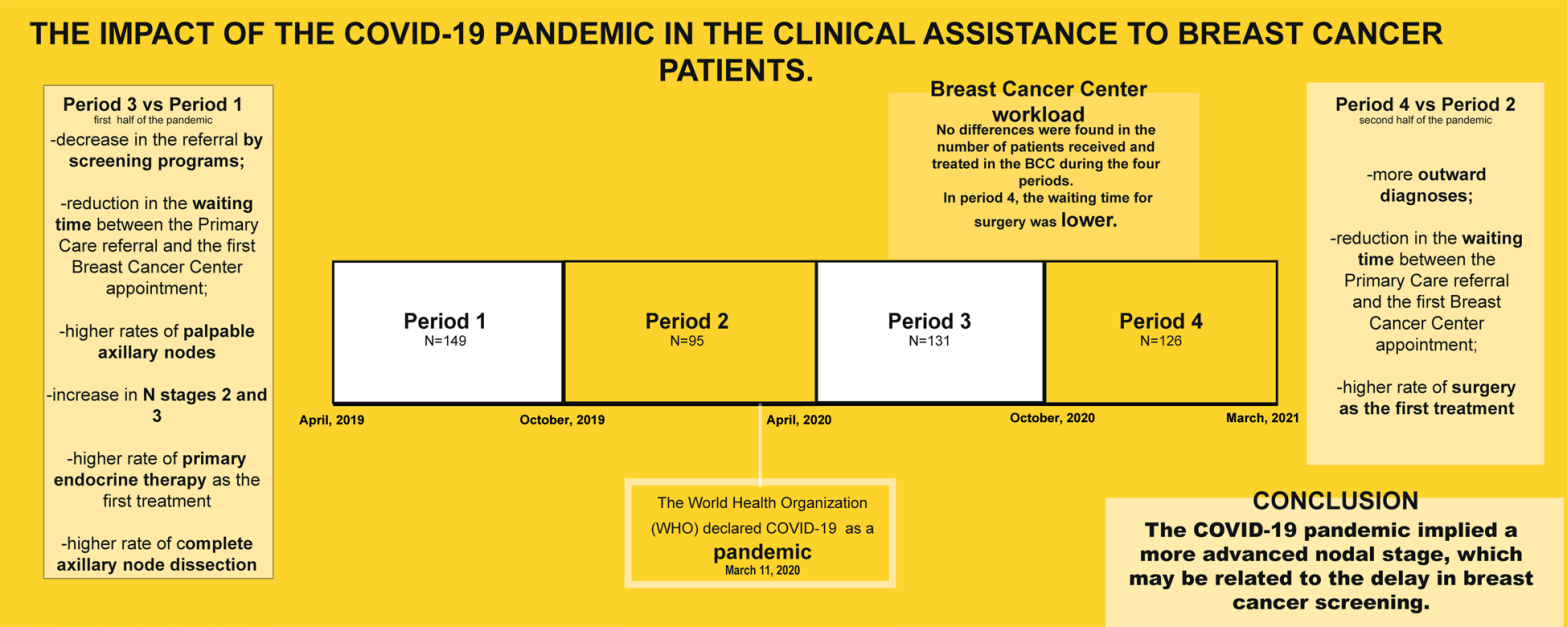

## Introduction

Breast cancer (BC) is the most common malignant tumor amongst Portuguese women; it also corresponds to the second cause of cancer-related death [1].

Over the past few years, due to screening programs and education for health, we verified a reduction in the stage of BC at diagnosis as well as an increase in the proportion of early-stage disease [2]. Biennially screening mammography is the most powerful tool available: if performed in women aged 50–69 years it reduces the mortality due to BC by 16.5% [3]. These facts substantiated an increase in overall survival [4] as well as an increase in breast-conserving surgery [5], that leads to better quality-of-life outcomes, especially in younger patients [6].

The emergence of a new disease resulting from the SARS-Cov2 infection had an undeniable impact all around the world. The World Health Organization (WHO) declared it as a pandemic on March 11, 2020. In Portugal, the first case was diagnosed on March 2, 2020 [7], and the Portuguese government decreed the first period of “state of emergency” on March 18, 2020 [8]. Portuguese Public Health Services (PPHS) adapted [9] to respond to the pandemic and to the rise in the demand for hospital care between these two dates. Hospital management boards outlined contingency plans, which included serious limitations to outpatient care, hospitalization, cancer treatments (chemotherapy and radiotherapy) and surgical treatments in the operating room [10]. Citizens were advised not to go to health institutions through the media unless it was compulsory [11].

Access to BC screening programs was interrupted and the private entities, which have contracts for providing breast imaging exams for the PPHS, also deeply reduced their operation [12].

These limitations to health care access, although alleviated, re-endured during the summer of 2020 and accentuated in autumn, with the development of the second pandemic wave, leading to the reduction of the number of breast pathology consultations [13] and mammograms [14]. Also, there is a perception among clinicians that more severe cases of breast cancer have manifested during the pandemic [15]. We postulate that these limitations to the access to health care have led to a worsening in the presentation of the breast malignant tumors.

The main aim of this study was to evaluate the impact of the COVID-19 pandemic on the new patients with breast cancer, considering the clinical presentation of the disease, the stage at diagnosis and the treatment strategy.

Secondary aim was to analyze the impact of the COVID-19 pandemic on the work volume of the breast cancer center (BCC) of the Centro Hospitalar Universitário São João (CHUSJ), particularly the numbers of out-patient consultations, patients treated, as well as breast cancer surgeries.

## Methods

### Type of study

This is a retrospective cohort study, comparing four consecutive sets of patients with breast cancer, treated at the BCC of CHUSJ, from April 1st, 2019 to March 31st, 2021.

**Fig. 1:**
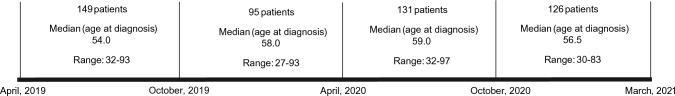
Distribution of the patients

The pandemic groups were compared with the homologous groups during the pre-COVID time. The distribution of the patients is displayed in Fig. 1.

Concerning our secondary aim, we compared the monthly figures in each period with the homologous period. The distribution of the data during each period is displayed in Table 3.

### Admission criteria

Patients over 18 years old with primary invasive or in situ breast cancer and with a first appointment in the BCC during the study period were eligible for our study.

To study BCC workload (the secondary aim of our study), patients with an appointment in the BCC during the study period were eligible.

### Exclusion criteria

Patients with secondary breast cancer and those with only one appointment in the BCC were excluded from our main objective analysis.

### Data collection

Cancer type was defined according to the International Classification of Disease 10th Revision (ICD-10). The American Joint Committee on Cancer (AJCC) Cancer Manual 8th Edition was used to staging the disease.

The variables collected for our main analysis were: age at diagnosis; dimension of the main lesion [clinically, (mm)]; dimension of the main lesion [mammography, (mm)]; dimension of the main lesion [ultrasound, (mm)], date of the referral by the primary care (PC); date of the first appointment at BCC; date of the first multidisciplinary meeting (MDM) to define treatment; referral method; outward diagnosis; cN; uN; tumour grade (OMS classification; needle biopsy); clinical stage; first treatment strategy; lymph node procedure.

For our secondary aim, we analyzed the number of MDM assessments; number of BCC appointments; number of patients on surgery waiting list; number of patients that entered surgery waiting list, number of patients on appointment waiting list, number of patients that entered appointment waiting list, waiting time for surgery and waiting time for the first appointment in the BCC.

### Statistics

Data management and statistical analysis were performed using IBM® SPSS® Statistics version 26.0. Categorical variables were expressed using counts and percentages, and continuous variables as means and standard deviation or as medians and range, as appropriate.

The chi-square test or Fisher’s test were used to assess the differences between groups. Continuous variables were analyzed using the median test. In all analyses, a *p*-value equal or below to 0.05 was considered statistically significant.

Missing data was not included in our analysis.

### Ethics

The study “The impact of the COVID-19 pandemic in the clinical assistance to breast cancer patients” was approved by the Ethics Committee of the CHUSJ on December 18th, 2020—CES 485-20.

### Confidentiality and data safety

Every case was designated by a serial number. Every study serial number was associated with a clinical file patient number. Only the principal investigator had access to that association database. Data was obtained retrospectively, retrieved from the patients' digital records and was further anonymized.

## Results

### Patients characteristics

In Period 1 we studied 149 patients, with a median age of 54 years (range: 32–93); in Period 2 there were 95 patients, with a median age of 58 years (range: 27–93 years); in Period 3 there were 131 patients with a median age of 59 years (range: 32–97 years); in Period 4 we analyzed 126 patients, with a median age of 56.5 years (range: 30–83 years) (Fig. 1). In the pre-pandemic Period we gathered 244 patients and in the pandemic period we collected 257 patients. There was no statistical difference in age between the groups.

The demographics and clinical variables are shown in Tables 1 and 2.

**Table 1:** Demographics

	Period 1	Period 2	Period 3	Period 4	Period 3 vs. Period 1	Period 4 vs. Period 2
	April to September of 2019 (*n* = 149)	October 2019 to March of 2020 (*n* = 95)	April to September of 2020 (*n* = 131)	October 2020 to March of 2021 (*n* = 126)		
	Median (range)	Median (range)	Median (range)	Median (range)	*p* value	*p* value
Age at diagnosis (years)^a^	54.0 (32–93)	58.0 (27–93)	59.0 (32–97)	56.5 (30–83)	0.95	0.508
Dimension of the main lesion [clinically, (mm)]^b^	20.0 (0–100)	20.0 (0–100)	20.0 (0–60)	20 (0–150)	0.939	0.739
Dimension of the main lesion [mammography, (mm)]^c^	20.0 (5–145)	23.0 (6–134)	21.0 (4–95)	19.0 (5–119)	0.312	0.019
Dimension of the main lesion [ultrasound, (mm)]^d^	20.0 (5–145)	23.0 (6–134)	21.0 (4–95)	19.0 (5–119)	0.177	0.019
Time between PC referral and first appointment (days)	12.0 (0–206)	11.0 (2–169)	6.0 (0–32)	6.0 (0–80)	< 0.001	0.009
Time between first appointment and first MDM assessment (days)	17.0 (0–138)	15.0 (0–269)	15.0 (0–174)	15.0 (0–181)	0.023	0.940

*PC* primary care, *MDM* multidisciplinary meeting

^a^1 missing in the Period 2

^b^18 missing in the period 1; 17 missing in the period 2; 24 missing in the period 3; 14 missing in the period 4

^c,d^1 missing in the Period 1; 2 missing in period 3 and 2 missing in the period 4

**Table 2:** Comparison by time period/time frame

	Period 1	Period 2	Period 3	Period 4	Period 3 vs. Period 1	Period 4 vs. Period 2
	April to September of 2019 (*n* = 149)	October 2019 to March of 2020 (*n* = 95)	April to September of 2020 (*n* = 131)	October 2020 to March of 2021 (*n* = 126)		
	*n* (%)	*n* (%)	*n* (%)	*n* (%)	*p* value	*p* value
Referral method^a^					0.002	0.368
Group A	114 (77.6)	78 (86.7)	117 (91.4)	98 (82.4)		
ER	3 (2.0)	3 (3.3)	2 (1.6)	2 (1.7)		
Screening program	30 (20.4)	9 (10.0)	9 (7.0)	19 (16.0)		
Outward diagnosis					0.600	0.003
Yes	8 (5.4)	5 (5.3)	9 (6.9)	24 (19.0)		
No	141 (94.6)	90 (94.7)	122 (93.1)	102 (81.0)		
cN^b^					0.001	0.517
0	134 (90.5)	66 (72.5)	94 (75.8)	94 (76.4)		
1–2	14 (9.5)	25 (27.5)	30 (24.2)	29 (23.6)		
uN^c^					0.050	0.058
0	113 (75.8)	64 (67.4)	95 (73.1)	95 (75.4)		
1	36 (24.2)	26 (27.4)	29 (22.3)	20 (15.9)		
2	0 (0.0)	4 (4.2)	5 (3.8)	11 (8.7)		
3	0 (0.0)	1 (1.1)	1 (0.8)	0 (0.0)		
Grade (needle biopsy)^d^					0.017	0.233
1	23 (15.5)	14 (14.9)	13 (10.1)	28 (22.6)		
2	72 (48.6)	44 (46.8)	48 (37.2)	46 (37.1)		
3	53 (35.8)	36 (38.3)	68 (52.7)	50 (40.3)		
Clinical stage^e^					0.407	0.149
0	18 (12.2)	9 (9.5)	15 (11.5)	13 (10.3)		
1	59 (39.9)	29 (30.5)	46 (35.4)	53 (42.1)		
2	65 (43.9)	47 (49.5)	56 (43.1)	45 (35.7)		
3	4 (2.7)	10 (10.5)	9 (6.9)	12 (9.5)		
4	2 (1.4)	0 (0.0)	4 (3.1)	3 (2.4)		
First treatment strategy					0.021	0.013
Endocrine therapy	9 (6.0)	25 (26.3)	21 (16.0)	15 (11.9)		
Chemo therapy	40 (26.8)	20 (21.1)	36 (27.5)	24 (19.0)		
Surgery	100 (67.1)	50(52.6)	74 (56.5)	87 (69.0)		
Lymph node first procedure^f^					0.030	0.402
None	12 (8.1)	14 (14.7)	18 (13.7)	22 (17.5)		
Sentinel node	119 (80.4)	61 (64.2)	87 (66.4)	86 (68.3)		
Axillary dissection	17 (11.5)	20 (21.1)	26 (19.8)	18 (14.3)		

Group A—Primary care doctor + random doctor + self-referral

*ER* emergency room

^a^17 missing ( 2 in the Period 1, 5 in Period 2, 3 in Period 3 and 7 in Period 4)

^b^15 missing (1 in Period 1; 4 in Period 2; 7 in Period 3 and 3 in Period 4)

^**c**^1 missing in Period 3

^d^6 missing (1 in Period 1; 1 in Period 2; 2 in Period 3; 1 in Period 4) and 1 undeterminable in Period 4

^e^2 missing (1 in period 1 and 1 in period 3)

^f^1 missing in Period 1

### Period 3 versus period 1 (April to September of 2020 vs. April to September 2019)

There was a decrease in the referral by cancer screening programs (7.0% vs. 20.4%) (*p* = 0.002). There were more palpable axillary nodes in the physical exam (cN) (24.2% vs. 9.5%) (*p* = 0.001). Ultrasound evaluation of the lymph nodes showed an increase in the uN2 (3.8% vs. 0.0%) and uN3 (0.8% vs. 0.0%) (*p* = 0.050). We found an increase in the proportion of Grade 3 tumors (52.7% vs. 35.8%; *p* = 0.017).

Concerning the first treatment, we observed a trend to an increase in the endocrine therapy (16.0% vs. 6.0%) and a decrease in the primary surgery (56.5% vs. 67.1%) (*p* = 0.021).

Regarding the first lymph node approach, there was a decrease in primary sentinel node biopsies (66.4% vs. 80.4%), at the expense of an increase in primary axillary lymph node dissections (19.8% vs. 11.5%) (*p* = 0.030).

We verified a strong reduction in the median waiting time for the first BCC appointment after PC referral (6.0 vs. 12.0 days; *p* < 0.001).

There were no statistical differences between the studied periods concerning the following variables: age at diagnosis, size of main lesion (clinically, mammography and ultrasound), outward diagnosis and clinical stage. The comparison between the two time periods are shown in Tables 1 and 2.

### Period 4 versus period 2 (October 2020 to March 2021 vs. October 2019 to March 2020)

There were more outward diagnoses in period 4 (19.0% vs. 5.3%; *p* = 0.003). There was an increase in surgery as first-line treatment (69.0% vs. 52.6%; *p* = 0.013). The median waiting time for the first BCC observation was reduced (6.0 vs. 11.0 days; *p* = 0.009). There were no statistical differences between the studied periods in the following variables: age at diagnosis, time between PC referral and first MDM discussion (days), time between first BCC appointment and first MDM discussion (days), time between first MDM assessment and first surgery, referral method, cN, uN, grade (needle biopsy) and clinical stage. The statistical results of the patients are shown in Tables 1 and 2.

### BCC workload

**Fig. 2:**
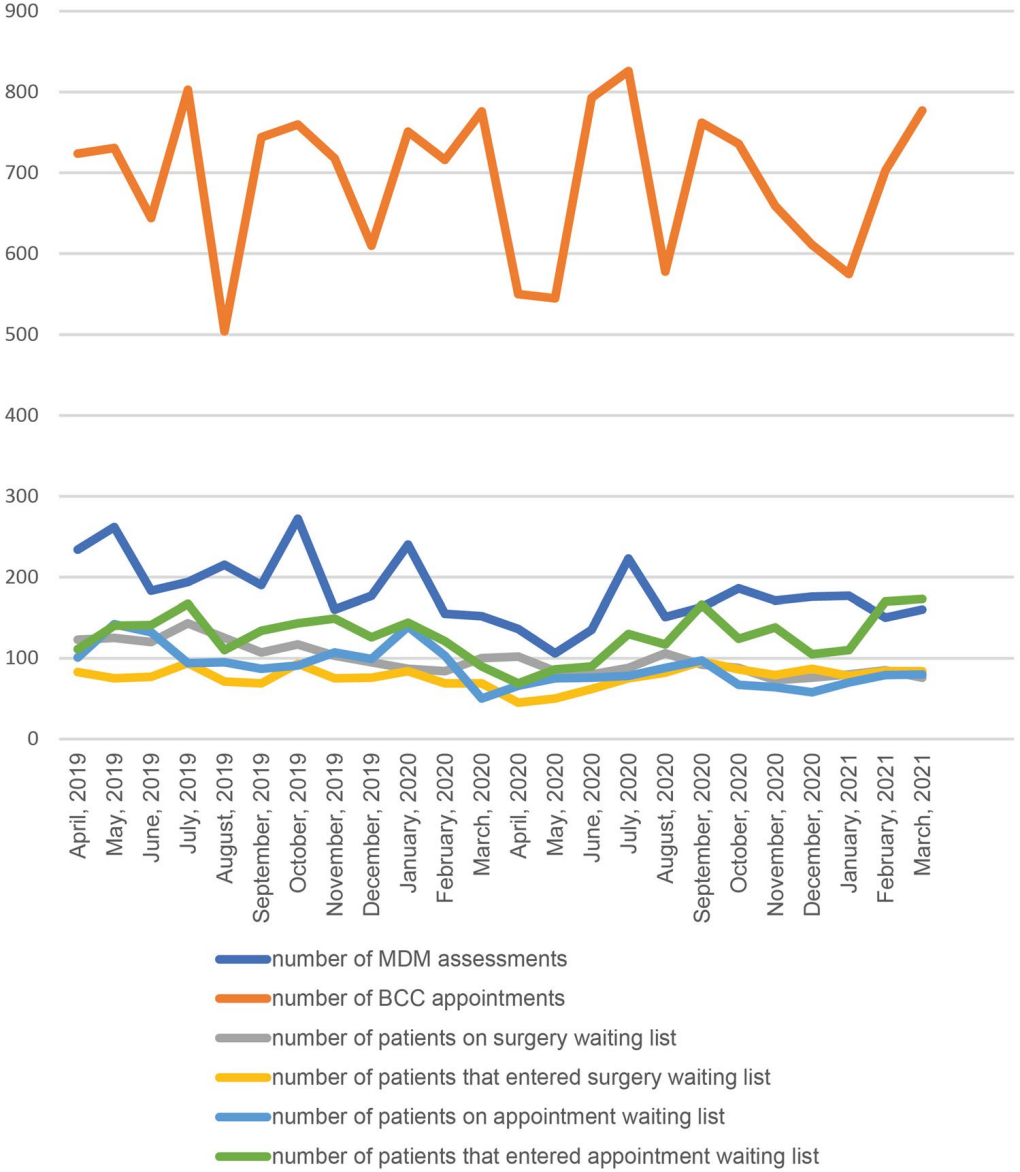
Work volume in the BCC. MDM multidisciplinary meeting, BCC breast cancer center

**Fig. 3:**
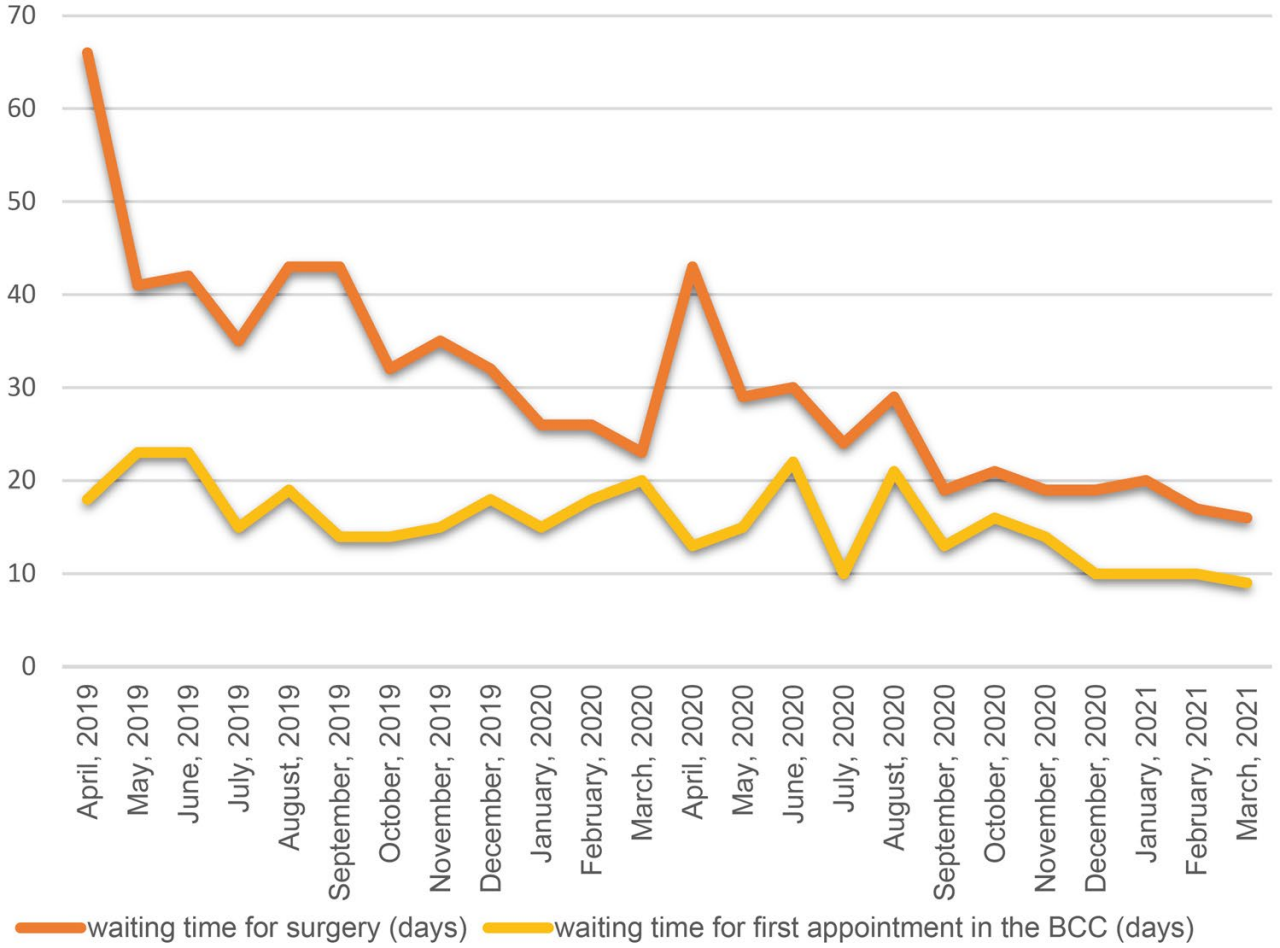
Median waiting time (days) for surgery and first appointment in the BCC. BCC breast cancer center

Results on the BCC workload are displayed in the Table 3 and Figs. 2, 3. In Period 4 (vs. Period 2), the median waiting time for surgery was reduced (*p* = 0.002), but no meaningful differences were observed in Period 3 (vs. Period 1). There were no statistical differences between the studied periods concerning the number of MDM assessments, BCC appointments, patients on surgery waiting lists, patients that entered surgery waiting lists, patients on appointment waiting lists, patients that entered appointment waiting lists and the median of waiting time for first appointment in the BCC.

**Table 3:** Breast cancer center workload during study time

Period	Month	Number of MDM assessments	Number of BCC appointments	Number of patients on surgery waiting list	Number of patients that entered surgery waiting list	Number of patients on appointment waiting list	Number of patients that entered appointment waiting list	Waiting time for surgery (median)	Waiting time for the first appointment in the BCC (median)
Period 1	April	234	724	123	83	101	111	123	18
	May	262	731	125	75	142	140	125	23
	June	183	644	120	77	132	141	120	23
	July	194	803	143	94	94	167	143	15
	August	215	504	125	71	95	110	125	19
	September	190	744	107	69	87	134	107	14
Period 2	October	272	760	117	93	91	143	117	14
	November	160	718	103	75	107	149	103	15
	December	177	610	95	76	99	126	95	18
	January	240	751	87	84	139	144	87	15
	February	155	716	84	69	103	121	84	18
	March	152	776	100	69	50	90	100	20
Period 3	April	136	550	102	45	66	69	102	13
	May	106	545	84	50	75	86	84	15
	June	135	793	80	62	76	90	80	22
	July	223	826	88	75	78	130	88	10
	August	151	578	106	82	88	117	106	21
	September	163	762	92	96	97	166	92	13
Period 4	October	186	736	88	86	67	124	88	16
	November	171	659	73	79	64	138	73	14
	December	176	611	76	87	58	105	76	10
	January	177	575	80	78	70	110	80	10
	February	150	703	85	84	79	170	85	10
	March	160	777	76	84	80	173	76	9
Period 3 vs. Period 1 (*p* value)		0.364	0.364	0.285	0.440	0.364	0.364	0.080	0.567
Period 4 vs. Period 2 (*p* value)		0.534	0.364	0.285	0.315	0.364	0.364	0.002	0.080

*MDM* multidisciplinary meeting, *BCC* breast cancer center

## Discussion

The screening program for breast cancer consists of a biennial mammogram, in asymptomatic women between the ages of 50 and 69 years. Breast cancer screening rates for women aged 50–69 years in Portugal are over 80% [16].

Nationally, since the implementation of the screening program in 1986, more than 4.3 million screening mammograms were performed (until 2020), which resulted in the early treatment of more than 20,000 women. Therefore, treatment was less aggressive and in some cases, it led to a total cure [17].

Due to the pandemic, in many countries, including Portugal, there was an interruption of breast cancer screening programs from March 16 to June 16 of 2020, which resulted in a lower number of mammograms being performed and subsequently, a lower number of potential tumors being detected. In fact, there was a reduction of 21% in the number of mammograms performed, which corresponded to an absolute value of 169.485 fewer women with a registered mammogram than in the 2 previous years (2019 vs. 2020) [18].

Owing to the pause in the screening programs, during Period 3 (April to September of 2020), we observed that the number of patients referred to the BCC by these programs diminished, while the percentage of patients referred by other health care specialists such as their Primary Care Doctor increased. Primary Care Doctors, other health specialists and self-referral (via emergency department) played an important role in the link between the patients and hospital care during the pandemic since the interruption of the screening program.

During the second period of the pandemic, Period 4, (October 2020 to March 2021), there were more outward diagnoses. This may be explained by the higher demand for private doctors during the pandemic, thanks to the preconceived idea of limited access to family doctors and to public hospital care. This finding is consistent with a study conducted in Bangladesh, which reported that approximately 75% of the responders sought diagnosis assistance from private hospitals during the pandemic [19].

For several weeks there were no referrals from the primary care. Consequently, there were no patients accumulated waiting for consultation. Thus, when patients started to get referred again, the time for the consultation was lower. This resulted in fewer days between referral by the PC and the first BCC consultation during both pandemic periods.

During Period 3, there were also fewer days between the first BCC appointment and the first MDM discussion. Since the workload of BCC lowered, a quicker response could be given to patients.

Although there was a higher percentage of patients being referred to the BCC by their Primary Care Doctor and other health specialists during the first half of the pandemic, patients were afraid of going to the hospital and leaving their homes. This fear might have contributed to some cancers not being diagnosed in time. A survey by the American College of Emergency Physicians demonstrated that nearly a third of patients (29%) had delayed or avoided seeking medical care due to the fear of contracting the coronavirus in March/April, 2020 [20]. In fact, COVID-19-related anxiety could also affect the patient’s decision-making process [21], leading to a lower number of patients seeking medical help and treatment.

The evaluation of axillary lymph nodes is an important factor in the management and staging of breast cancer as it is a predictor of survival outcome [22]. In the first pandemic period (Period 3), we found higher rates of palpable axillary nodes on physical examination and an increase in N stages 2 and 3. Ultrasound evaluation was in line with the clinical findings during the physical examination. Other recently published studies conducted in Italy demonstrated that a 2 month stop in mammographic screening produced a significant increase in node-positive breast cancer [23] and that during 2020 there was an increase in symptomatic patients being diagnosed [24] which is consistent with our results and with our hypothesis.

Since more suspected nodes were present at diagnosis, a higher number of needle biopsies were performed to characterize these nodes. If the biopsy confirmed lymph node metastasis, patients were submitted to lymph node dissection instead of a sentinel node biopsy [25]. Therefore, in juxtaposition with alternative research [26], we observed a lower number of sentinel node procedures and a higher number of lymph node axillary dissections because of the higher number of metastatic nodes at diagnosis.

To offload hospitals and better treat patients during the pandemic without compromising their health, many societies made extraordinary recommendations. One of these was to postpone surgery in favor of neoadjuvant endocrine therapy in early-stage ER-positive and HER-negative patients until regular workload could be resumed [27, 28]. This change in the treatment approach is consistent with the result that we observed with an increase in endocrine therapy, with a subsequent decrease in the primary surgery rates. A multicenter international analysis on the effect of the COVID-19 pandemic in EUSOMA-certified breast cancer centers (which includes BCC) showed that neoadjuvant treatment was used safely to delay surgery, although there was a significantly higher lymph node stage at presentation [29].

Due to the contingency plans implemented by hospital management boards, there were serious limitations on access to surgeries and oncologic treatments, which resulted in a lower rate of surgical treatments. In the USA, during the early stages of the global pandemic, breast cancer surgery declined significantly as well [30].

In the 4th period, there was a recovery in surgery as first-line treatment, and a decrease in chemotherapy and endocrine therapy, which might have resulted from the alleviation of the governmental limitations, leading to the return of normal surgical activity. Indeed, finding aligns with a recently published study that documents a complete recovery in number of breast surgeries performed since the second half of the lockdown period [31]

Patients also underwent surgery faster during this post-limitations period since there was a lowering in the absolute number of patients that were waiting for surgery, which resulted in a lower median time of waiting for surgery. It has been shown that there were statistically significant differences, favoring 2020, when analyzing time-to surgery and time-to radiotherapy [32]. While it is important to minimize unnecessary delays, the emphasis on breast cancer treatment should move away from striving to meet a specific time limit for surgery. Instead, the priority should be placed on ensuring that every aspect of patient’s care contributes effectively and optimally to their overall management [33].

One limitation of the present study is its retrospective design. In fact, multiple healthcare professionals were involved in patient care, which resulted in a less accurate and consistent database than the one that could be achieved with a prospective cohort study.

Treatment approaches were adapted rapidly due to the dedication and effort put into by the BCC team. Although at the beginning of the pandemic, most health care services stopped or slowed down their work volume, the BCC team was able to efficiently treat breast cancer patients without increasing waiting times. In fact, the effect of the COVID-19 pandemic on EUSOMA-certified breast cancer centers showed that the quality of breast cancer care was well maintained during the pandemic period [29].

## Conclusion

This study showed no impact of the COVID-19 pandemic on BC T stage at diagnosis. However, the presence of lymph node metastasis at diagnosis was more frequent than before and this resulted in differences in the treatment strategy. At the same time, the referrals from the screening program diminished significantly.

Considering the BCC work volume and waiting times, COVID-19 pandemic didn’t had a significant impact.

## Data Availability

Due to the nature of the research, due to [ethical/legal/commercial] supporting data is not available.
